# Feasibility of the TBDx automated digital microscopy system for the diagnosis of pulmonary tuberculosis

**DOI:** 10.1371/journal.pone.0173092

**Published:** 2017-03-02

**Authors:** Pamela Nabeta, Joshua Havumaki, Dang Thi Minh Ha, Tatiana Caceres, Pham Thu Hang, Jimena Collantes, Nguyen Thi Ngoc Lan, Eduardo Gotuzzo, Claudia M. Denkinger

**Affiliations:** 1 FIND, Geneva, Switzerland; 2 TB Department, Pham Ngoc Thach Hospital, Ho Chi Minh City, Vietnam; 3 Instituto de Medicina Tropical Alexander von Humboldt, Universidad Peruana Cayetano Heredia, Lima, Peru; Wadsworth Center, UNITED STATES

## Abstract

**Background:**

Improved and affordable diagnostic or triage tests are urgently needed at the microscopy centre level. Automated digital microscopy has the potential to overcome issues related to conventional microscopy, including training time requirement and inconsistencies in results interpretation.

**Methods:**

For this blinded prospective study, sputum samples were collected from adults with presumptive pulmonary tuberculosis in Lima, Peru and Ho Chi Minh City, Vietnam. TBDx performance was evaluated as a stand-alone and as a triage test against conventional microscopy and Xpert, with culture as the reference standard. Xpert was used to confirm positive cases.

**Findings:**

A total of 613 subjects were enrolled between October 2014 and March 2015, with 539 included in the final analysis. The sensitivity of TBDx was 62·2% (95% CI 56·6–67·4) and specificity was 90·7% (95% CI 85·9–94·2) compared to culture. The algorithm assessing TBDx as a triage test achieved a specificity of 100% while maintaining sensitivity.

**Interpretation:**

While the diagnostic performance of TBDx did not reach the levels obtained by experienced microscopists in reference laboratories, it is conceivable that it would exceed the performance of less experienced microscopists. In the absence of highly sensitive and specific molecular tests at the microscopy centre level, TBDx in a triage-testing algorithm would optimize specificity and limit overall cost without compromising the number of patients receiving up-front drug susceptibility testing for rifampicin. However, the algorithm would miss over one third of patients compared to Xpert alone.

## Introduction

Despite the introduction of Xpert MTB/RIF (Xpert; Cepheid, CA, US) -a more sensitive molecular test for the diagnosis of pulmonary tuberculosis (TB)- smear microscopy still plays a key role in many settings where challenging environmental conditions prevail and the cost of Xpert is considered a barrier. Smear microscopy, however, is labour-intense and requires extensive training for users to become proficient. Furthermore, results interpretation is often inconsistent due to variable operator proficiency and fatigue in high-throughput settings [1].

Automated digital microscopy uses predefined algorithms to identify TB-like organisms in sputum smear and may offer an opportunity to overcome the challenges of conventional smear microscopy, while preserving its high specificity and low cost. TBDx (Applied Visual Sciences [APVS] Inc., Virginia, US) is a fully automated fluorescence microscopy reading system that can detect TB within 5 minutes from Auramine-stained slides. A proof of concept study illustrated the potential of TBDx to replace conventional microscopy methods, achieving a sensitivity of 75·8% [2]. However, specificity was far below routine microscopy at 43·5% and required improvement. Since then, important software changes have been implemented. A more recent study in South Africa, comparing the performance of TBDx to culture given these software updates, showed 62% sensitivity and 99·7% specificity when used as a stand-alone test [3].

Here we report the accuracy of TBDx as a stand-alone diagnostic tool compared to culture and assess TBDx operational characteristics in laboratories with smear microscopy experience in two high-burden settings. We also assessed the use of TBDx as a triage tool in a diagnostic algorithm, with positive results being confirmed by Xpert. This diagnostic algorithm has the potential to increase specificity of automated microscopy while overall decreasing the cost of Xpert implementation.

## Materials and methods

### Study design

This was a blinded prospective study conducted at two reference laboratories in Lima, Peru and Ho Chi Minh City, Vietnam. Data were collected by local investigators. The study was conducted in accordance with the study protocol, which was approved by Institutional Review Boards at each site in September, 2014 (Universidad Peruana Cayetano Heredia in Peru; Pham Ngoc Thach Hospital and The Health Service of Ho Chi Minh City in Vietnam). Patients were recruited for this study between 7 October, 2014 and 11 March, 2015. Written informed consent was obtained from all participants prior to enrolment. The results of TBDx were not used to inform patient management.

### Participants

Consecutive adults (18 years of age or older) with presumptive pulmonary TB were recruited from 30 peripheral health centres in Lima in a district with high rates of TB case notification and a reference hospital in Ho Chi Minh City that serves as a referral centre for patients with lung diseases. Subjects who had received more than two doses of anti-TB therapy in the previous 60 days were excluded. Patients were required to provide at least 3 mL of sputum in order to participate in the study.

### Test methods

Eligible subjects were asked to provide two sputum samples. Specimens were collected and stored at 2–8°C before processing within 24 to 48h. Sputum samples were not heat-killed, but were directly homogenized with sterile glass beads and vortexed. Direct smears were prepared for light microscopy (Ziehl Neelsen [ZN] staining), fluorescent light-emitting diode (LED) microscopy and TBDx (Auramine-O staining) in a biosafety cabinet [4, 5]. Smear staining and reading for ZN and LED microscopy were performed in accordance with International Union Against Tuberculosis and Lung Disease (IUATLD) and WHO guidelines [4, 5]. The remaining sputum underwent standard *N*-acetyl-L-cysteine-NaOH (NALC-NaOH) decontamination [6]. The processed sample was then subjected to TBDx and LED microscopy, solid (Löwenstein-Jensen [LJ] media) and liquid culture (Mycobacteria Growth Indicator Tube [MGIT] 960 culture; BD Microbiology Systems) [7], as well as Xpert. The second sputum sample underwent NALC-NaOH decontamination followed by LJ and MGIT culture. All positive cultures underwent identification for *Mycobacterium tuberculosis* complex (MTB) using MPT64 antigen detection (Capilia TB, Tauns Laboratories, Shizuoka, Japan) [8]. Contaminated cultures were reprocessed to recover mycobacteria and the final results were reported.

### TBDx assay

TBDx is an automated microscopy solution with integrated hardware and software. The platform requires an Olympus BX41 microscope with a 40X objective lens that is fitted with a camera and an automated movable slide stage (with a 4-slide capacity). An attached computer receives high-quality digital images acquired from the camera.

TBDx was installed in the smear microscopy area of each laboratory by APVS. Two days of hands-on, onsite TBDx training was provided to laboratory technicians with prior experience using smear microscopy, followed by 1-day observation. Each operator performed 3–4 runs under supervision and a proficiency assessment was conducted prior to study initiation.

**Fig 1:**
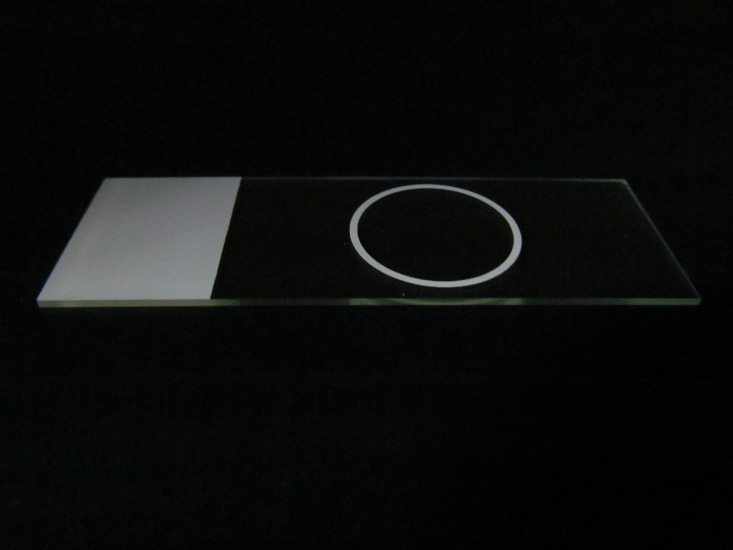
Target annotated slides. Target annotated slides provided by Applied Visual Sciences Inc. (distributed by Erez Medical Ltd., UK). The use of these slides allowed standardization of smear preparation.

Smears were prepared using customized glass slides provided by APVS. A delineated circular area of approximately 2 cm in diameter facilitates smearing, focusing and reading of the slides by TBDx (Fig 1).

TBDx was performed with a 4-slide stage following conventional Auramine-O staining procedures for smear preparation [4, 5]. The operator entered the sample identification in the computer and manually placed the slides on the movable stage for automated read-out of each slide as previously described [3]. Briefly, once the slide was placed on the microscope stage, TBDx automatically focuses the microscope, digitises 300 fields of view (FOV) at 40X magnification and downloads the data to the computer. The computer then uses proprietary algorithms to detect and count acid-fast bacilli-like organisms on the digitised FOV. Each of the 300 FOV are then individually analysed by TBDx, which provides specific counts of TB-like organisms. Although the TBDx results were automatically recorded, the laboratory technicians operating the TBDx were blinded to conventional smear and Xpert results. Importantly, the technicians processing conventional smear microscopy were also blinded to all other results.

Operational characteristics and ease-of-use of the TBDx system were evaluated at the end of the study using a standard questionnaire to assess the test as an alternative to conventional fluorescence microscopy.

### Analysis

Subjects were divided into two categories for analysis: those with culture-positive pulmonary TB, defined as those patients with positive results for MTB on at least one of four cultures, and those patients who were TB-negative, having no microbiological evidence of pulmonary TB infection (smear-negative, Xpert-negative and culture-negative). A smear-positive case was defined as a patient with at least one of four smears of scanty or higher grade per IUATLD scores for either ZN or LED [9]. Samples for which cultures yielded non-tuberculous *mycobacteria* (NTM), as well as smear-positive samples with negative cultures, were included in a sub-analysis. As for a cut-off to define TBDx results, we used the same scenarios considered by Ismail *et al*. [3] to interpret a TBDx positive result: at least one AFB, more than one AFB, and at least 10 AFB in 300 FOV, for ease of comparison. Additionally, a receiver operating characteristic (ROC) curve was generated from TBDx quantitative results.

### Registration

This study was retrospectively registered at clinicaltrials.gov (NCT02912832). Clinical trial registration was delayed, as investigators did not have a trial registration policy in place at the time of this study. The authors confirm that all ongoing and related trials for this intervention are registered.

## Results

### Study subjects and sample characteristics

**Fig 2:**
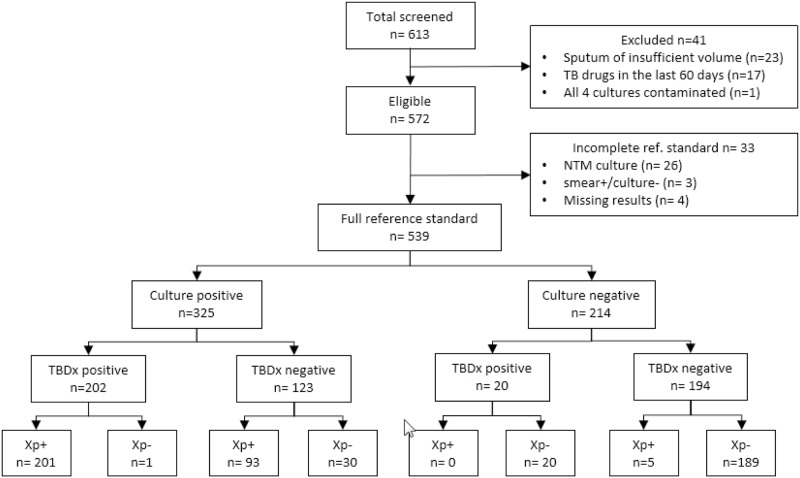
Study flowchart. Abbreviations: TB: tuberculosis; NTM: non-tuberculous mycobacteria; Xp: Xpert MTB/RIF.

A total of 613 subjects with symptoms suggestive of pulmonary TB were recruited between October 2014 and March 2015 in Peru (310 recruited) and Vietnam (303 recruited). Forty-two participants were excluded based on predefined exclusion criteria (Fig 2). Among 572 eligible subjects, 33 did not have complete reference standard results available. Of these twenty-six only had culture results positive for NTM, three were smear-positive but culture-negative and four were missing results and were excluded from the main analysis. Of the 539 subjects available for analysis, 269 were from Peru and 270 were from Vietnam. The mean age was 36 years (range: 18–93 years), 63% male. Among subjects included in the main analysis, 325 (60·3%) were culture-confirmed TB cases and 214 (39·7%) had no microbiological evidence of TB. Xpert was performed on the corresponding 539 sample pellets and was positive in 299 (sensitivity 90·5%, 95% CI 86·7–93·4), and negative in 240 (specificity 97·7% 95% CI 94·6–99·2) (Fig 2).

### Performance of direct conventional microscopy against culture

Among the 325 culture-positive cases, 264 were smear-positive by LED (sensitivity 81·2%, 95% CI 76·6–85·3). Among the 214 culture-negative cases, all were smear-negative by LED (specificity 100%, 95% CI 98·3–100). Similarly, 260 of the culture-positive cases were smear-positive (sensitivity 80·0%, 95% CI 75·2–84·2) while all culture-negative cases were smear-negative by ZN (specificity 100%, 95% CI 98·3–100) (Table 1).

**Table 1:** Diagnostic performance of LED, ZN and TBDx from direct sputum samples compared to culture* from two samples.

		TP (n)	FN (n)	FP (n)	TN (n)	Sensitivity (95% CI)	Specificity (95% CI)
**LED**		264	61	0	214	81·2% (76·6%- 85·3%)	100% (98·3%-100%)
**ZN**		260	65	0	214	80% (75·2%-84·2%)	100% (98·3%-100%)
**TBDx**	≥1 AFB	202	123	20	194	62·2% (56·6%-67·4%)	90·7% (85·9%-94·2%)
	>1 AFB	184	141	10	204	56·6% (51%-62·1%)	95·3% (91·6%-97·7%)
	≥10 AFB	144	181	0	214	44·3% (38·8%-49·9%)	100% (98·3%-100%)

Abbreviations: LED: fluorescent light-emitting diode microscopy; ZN: light microscopy Ziehl Neelsen staining; AFB: acid-fast bacilli; TP: true positive; FN: false negative; FP: false positive; TN: true negative

*Composed of two solid (Löwenstein Jensen) and two liquid (MGIT) cultures

### TBDx as a stand-alone diagnostic test

TBDx, performed on direct sputum, detected 202 out of 325 culture-positive cases when ≥ 1 AFB was considered as positive (62·2% sensitivity, 95% CI 56·6–67·4). The sensitivity was 74·2% (95% CI 68·5–79·4) for direct LED-positive, culture-positive cases. Among culture-negative cases, TBDx correctly classified 194 out of 214 (90·7% specificity, 95% CI 85·9–94·2). The specificity was comparable (90·6%, 95% CI 85·6–94·1) for direct LED-negative, culture-negative cases. TBDx, performed on concentrated sputum, detected 223 out of 325 culture-positive cases (68·6% sensitivity, 95% CI 63·3–73·6), and correctly classified 192 out of 214 culture-negative cases (89·7% specificity, 95% CI 84·8–93·4). The difference in sensitivity when comparing the performance of TBDx using direct and concentrated smears was statistically significant (p-value = 0·01 McNemar Chi squared test). The sensitivity was significantly lower in Vietnam for both direct (51% vs. 79·5% in Peru, p<0·0001) and concentrated (64·1% vs. 75·6% in Peru, p = 0·024) smears, though specificity was similar between sites.

**Fig 3:**
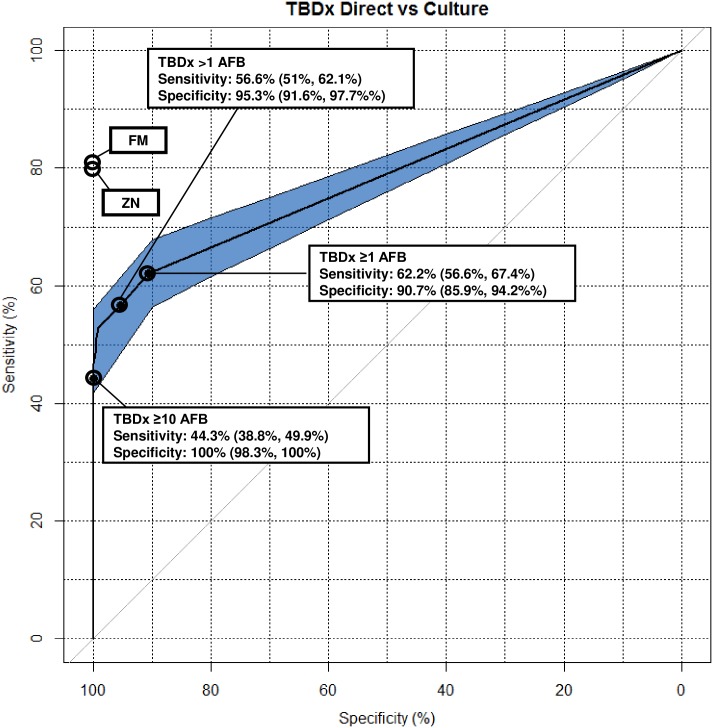
ROC curve for different TBDx cut-off points. Abbreviations: AFB: acid-fast bacilli; FM: fluorescence microscopy; ZN: light microscopy.

The specificity of TBDx improved considerably, overall, with an algorithm asking for at least 10 AFB identified for a result to be called positive. However, sensitivity decreased substantially (sensitivity fell from 62·2%, 95% CI 56·6–67·4% with ≥ 1 AFB, to 44·3%, 95% CI 38·8–49·9% with ≥10 AFB). A cut-off asking for more than 1 AFB for a result to be called positive achieved a sensitivity of 56·6% (95% CI 51–62·1%) and specificity of 95·3% (95% CI 91·6–97·7%). These results were considerably lower than those of an experienced microscopist using LED microscopy (sensitivity 81·2%, CI 76·6–85·3%; specificity 100%, 95% CI 98·3–100%). More detailed results, in form of a ROC curve, are shown in Fig 3.

### TBDx as a triage test

The performance of the algorithm combining direct TBDx as a triage test, followed by Xpert for confirmation of positive TBDx cases for the different cut-off points, is shown in Table 2. If TBDx cases with ≥ 1 AFB were considered positive, all false-positive results would be eliminated and a specificity of 100% would be achieved while maintaining a sensitivity of 62%. Such an algorithm would require only 201 Xpert cartridges in this high prevalence setting (saving 338 cartridges), although 31·6% cases would be missed (93/294) in comparison to a scenario where all patients would receive Xpert testing upfront independent of smear status. If a higher number of AFB would be required for TBDx to be called positive, the specificity would be similarly improved with the addition of Xpert; however, more cases would be missed (TBDx false negative) in comparison to an Xpert-only algorithm (Table 2).

**Table 2:** Diagnostic performance of a triage-testing algorithm including TBDx followed by Xpert MTB/RIF on TBDx positive results from direct sputum samples.

	TP (n)	FN (n)	FP (n)	TN (n)	Sensitivity (95% CI)	Specificity (95% CI)	PPV	NPV	Xpert carts. used
**≥1 AFB**	201	124	0	214	61·8% (56·3%-67·2%)	100% (98·3%-100%)	100%	63·3%	201
**≥10 AFB**	144	181	0	214	44·3% (38·8%-49·9%)	100% (98·3%-100%)	100%	54·2%	144
**Xpert**	294	31	5	209	90·5% (86·7%-93·4%)	97·7% (94·6%-99·2%)	98·3%	87·1%	539

Abbreviations: AFB: acid-fast bacilli; TP: true positive; FN: false negative; FP: false positive; TN: true negative; PPV: positive predictive value; NPV: negative predictive value

### NTM and smear-positive, culture-negative sub-analysis

Of the 26 NTM cases identified, one was excluded from the sub-analysis due to invalid TBDx result likely due to the system not being able to focus. Of the remaining 25 NTM cases, 23 (92%) were TBDx-negative of which 20 (87%) were LED-negative on direct smear. The two (8%) NTM and TBDx-positive cases were also LED-positive. Additionally, three LED-positive, culture-negative cases were all reported as negative by TBDx. If NTM cases were included in the overall analysis, the specificity of the direct TBDx would only minimally changed (from 90·7% to 90·8% specificity; 95% CI 86·4%, 94·1%).

### Invalid results and ease-of-use assessment

The overall TBDx failure rate was 5·9% (36/613) on direct samples, though 34 of the 36 failed samples had valid results upon re-reading (0·3% failure rate after re-reading). No invalid results were reported with LED. Xpert had an invalid rate of 0·8% (5/613), though 4 of the 5 invalid samples with sufficient volume were repeated with valid results (0·2% invalid rate after repeat). The culture contamination rates were 1·7% (21/1206) and 2·3% (28/1207) for liquid and solid culture, respectively.

Four laboratory technicians and two laboratory managers with average of seven years of experience in smear microscopy, completed the questionnaire to assess the ease-of-use and perceived use of TBDx as an alternative to fluorescence microscopy (Table 3).

**Table 3:** Ease-of-use and suitability assessment.

Topic	Multiple choice	Agreement
First use of TBDx	Self-explanatory, can be done without reading the user manual	0%
	Easy, but a user manual with instructions is required	50%
	Rather difficult; some problems were faced during installation/first use	50%
	Very difficult; cannot be expected without on-site training	0%
Training requirements for microscopist	1 day	33%
	2–3 days	50%
	4 or more days	17%
User manual satisfaction	Easy to read and understand; covers all questions I had during installation/use/troubleshooting	100%
	Most sections easy to read and understand, with some weaknesses	0%
	Rather cumbersome to read (information required is not found easily; not enough pictures that allow understanding at first glance)	0%
Contrast, colour, background satisfaction	Very satisfied	50%
	Satisfied	50%
	Not satisfied	0%
Ease of sample information capture	Yes, always	67%
	Yes, most of the times	17%
	No	0%
	Don’t know/NA	17%
Ease of Z working range level setup	Easy	33%
	Difficult, but only a matter of training	67%
	Very difficult	0%
Ease of use compared to LED	More difficult	67%
	Same	33%
	Less difficult	0%
	Don’t know/NA	0%
Hands-on time compared to LED	More time	100%
	Same	0%
	Less time	0%
	Don’t know/NA	0%
Hands-on time for negative smears	More time	83%
	Same	17%
	Less time	0%
	Don’t know/NA	0%
Use of TBDx as an alternative to LED	Yes	50%
	No	33%
	Don’t know/NA	17%
Main barriers for implementation	Total time	20%
	Complexity	0%
	Low specificity	20%
	Number of steps	30%
	Microscope-specific	30%
	Waste management	0%

Questionnaire applied to 4 laboratory technicians and 2 laboratory managers in Peru and Vietnam (2 to 11 years of experience in smear microscopy)

Overall, participants were satisfied with TBDx technical features such as contrast, colour and background of the images and sample information entry in the system. Although the user manual was considered easy to read and understand by all participants, some issues were faced during installation (the power source was damaged at the site in Peru due to difference in voltage). Setting the Z working range level (Z axis for image focusing) was considered difficult, and this was something that had to be done each day, but was ultimately considered to be only a matter of training. The overall hands-on time, particularly for negative smears, was perceived to be longer with TBDx compared to LED for all participants. Regarding the use of TBDx as an alternative to LED, half of the participants were in favour of TBDx. Participants indicated the most common potential barriers for implementation: microscope-specific system (30%), the number of steps to perform a test (30%), the total assay time (20%) and the low specificity (20%).

## Discussion

The performance of TBDx as a stand-alone diagnostic tool, compared to culture, was lower than that of conventional microscopy performed by experienced microscopists in this two-centre study. TBDx specificity approached that of LED if a cut-off of ≥ 10 AFB was set, although sensitivity was negatively affected (almost half of that of conventional microscopy). The specificity of TBDx, given more sensitive cut-offs (e.g. ≥AFB 1) in routine reference settings, with a prevalence in the range of 20–30%, would result in substantial overtreatment given a positive predictive value of only 62·6%-74·1%. In more decentralized settings, where TB prevalence ranges around 5–10%, the positive predictive value of TBDx would be even lower (26%-42·6%) and the amount of overtreatment even more substantial.

Currently, conventional microscopy prevails as the primary diagnostic test in a large number of high-burden countries, despite the WHO recommending Xpert as the first test for the diagnosis of TB [10, 11]. The primary reason for the limited rollout of Xpert has been resource constraints. While TBDx is not yet widely available, the anticipated cost of TBDx is as low as $3.35 per test [12], although higher than conventional smear-microscopy, it will considerably reduce the cost of diagnosis compared to Xpert which would make it an attractive triage test [13]. While TBDx did not perform at the same sensitivity as conventional smear microscopy in this study, it is conceivable that it would exceed the performance of smear in many settings, given that there are many less experienced microscopists currently performing conventional microscopy in resource-limited settings [14]. The performance of smear microscopy is highly dependent on the operator’s expertise and the time available to examine a smear, which under routine conditions in high burden settings with limited staff could yield sensitivity fluctuations from 20% to 80% [15]. Moreover, results from external quality assessments of smear microscopy suggest that most errors occur due to false-negative results [14] suggesting that under routine conditions the reading time may be reduced. In this context, TBDx would possibly allow for consistency in results though more multisite studies will be required to confirm this. Furthermore, smear proficiency is difficult to maintain where the staff turnover is high [14]. Based on feedback from users in our study, TBDx could be potentially implemented with targeted training to focus on difficult steps such as the Z working range level setup.

Additional benefits of TBDx include the ability to automatically assess a high number of slides in high-throughput settings, particularly when integrated with a 200-slides stage with automated slide loader. Though not assessed in this study, this automated feature may be useful for quality assurance by reference laboratories [14]. Another possible advantage of automated microscopy over conventional microscopy is the ability to transmit results in real-time directly from the instrument by utilizing connectivity, thus enabling many different options for use, from directly informing the patients of results to surveillance as well as device and user management [16]. However, the barriers to TBDx implementation, including the use of dedicated slides for smear standardization, use of a specific microscope (i.e. Olympus) and perceived long hands-on time cannot be underestimated when considering TBDx as a replacement for conventional microscopy methods.

This study found the current TBDx system to have inadequate diagnostic performance as a stand-alone tool, but in the absence of a more sensitive and specific molecular test that could be implemented at a microscopy centre level, a triage-testing algorithm could be considered in some (limited) settings [17], and potentially overcome the barriers related to the larger implementation of Xpert. In a triage-testing algorithm, TBDx specificity was optimized to that of Xpert (and similar to that of conventional microscopy), while the overall cost of Xpert implementation was reduced compared to the algorithm relying only on Xpert testing. However, it would have to be accepted that sensitivity would be lower than of an Xpert-only implementation, and about one third of TB cases would be missed. Such an algorithm would also limit the number of up-front rapid drug-susceptibility tests, independent of risk factors, which is of particular importance in high MDR-settings and not in line with the WHO’s End-TB Strategy recommendation [18, 19].

The strengths of our study include the use of a rigorous gold standard, composed of four cultures, and comparison of direct and concentrated smears. Whereas the assessment of TBDx by highly experienced microscopists from reference laboratories can be considered a strength of this study, the applicability of results may be restricted, as the sensitivity of conventional smear microscopy was higher than expected [15, 20]. Additionally, the culture positivity rate (60·3%) was higher than expected in this study, likely due to the study population including a large number of referral cases (Vietnam) and patients from a region with a high TB case detection rate (Peru). These patients were more likely to have active TB infections, and so these results may differ from those obtained in routine clinical settings. Other limitations include the low HIV prevalence in the study population, as the assessments of false-negative rates for the different methods may not be representative of populations with high HIV rates [21], and the use of Xpert MTB/RIF on concentrated samples in this study, which has been previously associated with slightly lower sensitivity on smear-negative samples [22].

Additional studies will be required to assess TBDx as an alternative to conventional microscopy in settings where microscopists are less experienced and where quality assurance is not available. Moreover, the ability to perform treatment monitoring using TBDx has not been evaluated in this study, or in other studies to date. This will need to be considered and evaluated should TBDx or any automated microscopy systems replace conventional microscopy [13]. The implementation of such a test should also consider the near-term availability of novel TB tests on molecular platforms such as the *Cepheid Omni* or possibly *Alere q* that are designed for implementation in decentralized settings such as microscopy centres [23]. On the other hand, these tests will not likely replace conventional microscopy methods unless they can be used for treatment monitoring, which would require special processing to avoid detection of dead bacteria in samples [24], and unless they can reach price points that would make large-scale implementation affordable to resource-limited countries [25].

Further improvement of the current TBDx version is necessary in order increase the likelihood of adoption. This may be achieved by simple adjustments, such as increasing the number of times the system focuses images to reduce variation due to smear quality, and confirming TBDx performance parameters are at least in line with LED- the current WHO-recommended TB diagnostic smear test. Although automated microscopy appears to be an attractive tool to overcome the challenges of operator variability of conventional smear microscopy, the potential value of TBDx as a triage test will depend on the willingness to pay and use the technology, the level of drug-resistance in the target population, as well as the access to more sensitive molecular tests at the microscopy centre level.

## Supporting information

S1 Table STARD checklist for TBDx evaluation. (DOCX)

S1 Protocol TBDx feasibility protocol. (PDF)

S1 Dataset TBDx user appraisal dataset. (XLSX)

S2 Dataset TBDx diagnostic performance evaluation dataset. (XLSX)

## References

[1] Chang J, Arbelaez P, Switz N, Reber C, Tapley A, Davis JL, et al Automated tuberculosis diagnosis using fluorescence images from a mobile microscope. *Med Image Comput Comput Assist Interv* 2012; 15(Pt 3): 345–52. 23286149 10.1007/978-3-642-33454-2_43 PMC3565532

[2] Lewis JJ, Chihota VN, van der Meulen M, Fourie PB, Fielding KL, Grant AD, et al "Proof-of-concept" evaluation of an automated sputum smear microscopy system for tuberculosis diagnosis. *PloS one* 2012; 7(11): e50173 10.1371/journal.pone.0050173 23209666 PMC3510232

[3] Ismail NA, Omar SV, Lewis JJ, Dowdy DW, Dreyer AW, Meulen H, et al Performance of a Novel Algorithm Using Automated Digital Microscopy for Diagnosing Tuberculosis. *Am J Respir Crit Care Med* 2015; 191(12): 1443–9. 10.1164/rccm.201502-0390OC 25826383

[4] Lumb R, Van Deun A, Bastian I, Fitz-Gerald M. Laboratory Diagnosis of Tuberculosis by Sputum Microscopy, The Handbook; 2013.

[5] World Health Organization. Tuberculosis laboratory biosafety manual. Geneva: World Health Organization, 2012. 24404640

[6] Kent PA, Kubica GP. Public health mycobacteriology—a guide for the level III laboratory. In: Services UDoHaH, editor.: Public Health Services, Centers of Disease Control; 1985.

[7] Siddiqi SH, Rüsch-Gerdes S. MGIT For BACTEC MGIT 960 TB System. 2006.

[8] Hillemann D, Ruesch-Gerdes S, Richter E. Application of the Capilia TB assay for culture confirmation of Mycobacterium tuberculosis complex isolates. *Int J Tuberc Lung Dis* 2005; 9(12): 1409–14011. 16466066

[9] World Health Organization. Definitions and reporting framework for tuberculosis—2013 revision—2014 update, 2014.

[10] World Health Organization. Policy update: Xpert MTB/RIF assay for the diagnosis of pulmonary and extrapulmonary TB in adults and children, 2013. 25473701

[11] World Health Organization. Xpert MTB/RIF implementation manual: technical and operational ‘how-to’; practical considerations. 2014. 25473699

[12] World Health Organization. High-priority target product profiles for new tuberculosis diagnostics: report of a consensus meeting. Geneva: World Health Organization, 2014.

[13] Jha S, Ismail N, Clark D, Lewis JJ, Omar S, Dreyer A, et al Cost-Effectiveness of Automated Digital Microscopy for Diagnosis of Active Tuberculosis. *PLOS ONE* 2016, 11(6): e0157554 10.1371/journal.pone.0157554 27322162 PMC4913947

[14] Van Rie A, Fitzgerald D, Kabuya G, Van Deun A, Tabala M, Jarret N, et al Sputum smear microscopy: evaluation of impact of training, microscope distribution, and use of external quality assessment guidelines for resource-poor settings. *J Clin Microbiol* 2008; 46(3): 897–901. 10.1128/JCM.01553-07 18174302 PMC2268372

[15] Steingart KR, Henry M, Ng V, Hopewell PC, Ramsay A, Cunningham J, et al Fluorescence versus conventional sputum smear microscopy for tuberculosis: a systematic review. *Lancet Infect Dis* 2006; 6(9): 570–81. 10.1016/S1473-3099(06)70578-3 16931408

[16] World Health Organization. National eHealth Strategy Toolkit. Geneva; 2012.

[17] Denkinger CM, Dolinger D, Schito M, Wells W, Cobelens F, Pai M, et al Target product profile of a molecular drug-susceptibility test for use in microscopy centers. *J Infect Dis* 2015; 211 Suppl 2: S39–49. 25765105 10.1093/infdis/jiu682 PMC4425821

[18] World Health Organization. Global Tuberculosis Control: WHO report 2014. Geneva: World Health Organization, 2014.

[19] World Health Organization. The End TB Strategy, 2015.

[20] Steingart KR, Henry M, Ng V, Hopewell PC, Ramsay A, Cunningham J, et al Fluorescence versus conventional sputum smear microscopy for tuberculosis: a systematic review. Lancet. 2006; 6: 570–581. 10.1016/S1473-3099(06)70578-3 16931408

[21] Cavanaugh JS, Modi S, Musau S, McCarthy K, Alexander H, Burmen B, et al Comparative yield of different diagnostic tests for tuberculosis among people living with HIV in Western Kenya. *PLoS One* 2016; 11(3):e0152364 10.1371/journal.pone.0152364 27023213 PMC4811572

[22] Steingart KR, Schiller I, Horne DJ, Pai M, Boehme CC, Dendukuri N. Xpert(R) MTB/RIF assay for pulmonary tuberculosis and rifampicin resistance in adults. *Cochrane Database Syst Rev* 2014; 1: CD009593. 10.1002/14651858.CD009593.pub3 PMC4470349 24448973

[23] Cepheid. World's Most Portable Molecular Diagnostics System Unveiled at AACC. 2015.

[24] Miotto P, Bigoni S, Migliori GB, Matteelli A, Cirillo DM. Early tuberculosis treatment monitoring by Xpert(R) MTB/RIF. *Eur Respir J* 2012; 39(5): 1269–71. 10.1183/09031936.00124711 22547737

[25] Pantoja A, Kik SV, Denkinger CM. Costs of novel tuberculosis diagnostics—will countries be able to afford it? *J Infect Dis* 2015; 211 Suppl 2: S67–77. 25765108 10.1093/infdis/jiu820

