# Supplementary material for: Feasibility of the TBDx automated digital microscopy system for the diagnosis of pulmonary tuberculosis
Source: PLoS One. 2017 Mar 2;12(3):e0173092. doi: 10.1371/journal.pone.0173092 (PMC5333855; doi:10.1371/journal.pone.0173092)
Supplement: S1 Protocol — (PDF) [file pone.0173092.s002.pdf]

STUDY PROTOCOL

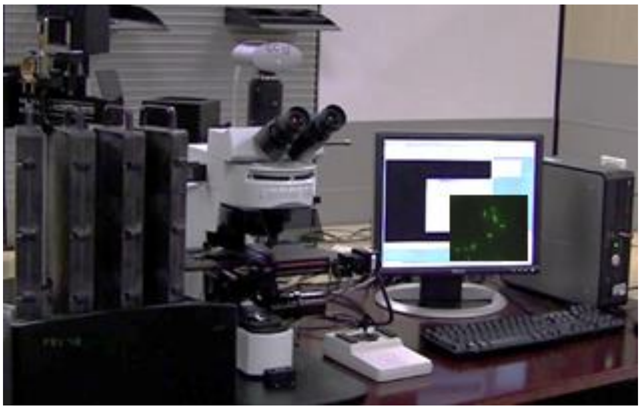

TBDx Feasibility Study

Prospective study to determine the feasibility of automated smear microscopy

Version and date: Version 3.0, 01 June 2014
Project and study: Clinical Study Platform/ 7012
Trial sites: Vietnam, Peru

Head of TB Program:
Dr Claudia Denkinger
Email:
claudia.denkinger@finddiagnostics.org

Project Manager:
Dr Pamela Nabeta
Email:
pamela.nabeta@finddiagnostics.org

Partnering for better diagnosis for all

Technical and Financial Agency:
FIND, Avenue de Budé 16, 1202 Genève, Switzerland   Tel: +41 22 710 0590   Fax: +41 22 710 0599   www.finddiagnostics.org

CONFIDENTIALITY STATEMENT

The information contained in this document, especially unpublished data, is the property of FIND (or under its control) and may not be reproduced, published or disclosed to others without written authorization.

Table of Content

1 INVESTIGATORS 4
1.1 Principal Investigator(s): 4
1.2 Co-Investigator(s): 4
2 STATEMENT OF PRINCIPAL INVESTIGATOR(S) 5
4 BACKGROUND 6
5 PURPOSE OF THE STUDY 6
6 STUDY OVERVIEW 6
7 PRODUCT DESCRIPTION 6
8 STUDY OBJECTIVES 7
9 STUDY DESIGN 7
9.1 Sample Size 8
9.2 Study Timelines & Deliverables 8
9.3 Study population 8
9.4 Subject recruitment 8
9.5 Sample processing 9
10 TBDXTEST PROCEDURE 9
10.1 Laboratory Requirements 10
10.2 Maintenance 10
10.3 Waste Procedures 10
10.4 Methods to be compared, description of reference standard 10
10.5 Minimization of bias 10
11 SUPPLY AND INVENTORY CONTROL 10
11.1 Shipments - Import and export permit needs 11
12 DATA MANAGEMENT AND QA 11
13 STUDY QA 12
13.1 Trial Site Certification and Proficiency Testing 12
13.2 Training, monitoring and auditing 12
14 DATA ANALYSIS 12
15 STUDY ETHICS 13
15.1 IRB and approval procedure 13
15.2 Possible benefits associated with the study 14
15.3 Risks associated with the study 14
15.4 Subject identification and confidentiality 14
15.5 Confidentiality of study documents and subject records 14
FIND APPROVAL 15

1 INVESTIGATORS

1.1 Principal Investigator(s):

Dr. Ngoc Lan Nguyen Thi
Pham Ngoc Thach Tuberculosis and Lung Disease Hospital (14)
120 Hung Vuong Street, District 5
Ho Chi Minh City, Vietnam
Tel: +84 839 574 369
ngoclan0456@yahoo.com

Dr. Eduardo Gotuzzo
Instituto de Medicina Tropical Alexander von Humboldt
Universidad Peruana Cayetano Heredia (01)
Av. Honorio Delgado 430 San Martin de Porres
Lima, Peru
Tel: +511 482-3910
eduardo.gotuzzo@upch.pe

1.2 Co-Investigator(s):

Dr. Ha Dang Thi Minh
Pham Ngoc Thach Tuberculosis and Lung Disease
Hospital (14)
120 Hung Vuong Street, District 5
Ho Chi Minh City
Vietnam
phadtm@oucru.org

Dr. Carlos Zamudio
Instituto de Medicina Tropical Alexander von
Humboldt
Universidad Peruana Cayetano Heredia (01)
Av. Honorio Delgado 430 San Martin de Porres
Lima, Peru
carlos.zamudio@upch.pe

2 STATEMENT OF PRINCIPAL INVESTIGATOR(S)

In signing this page, I agree to conduct the study in accordance with the relevant, current protocol, applicable regulations and institutional policy.

I will ensure that the requirements relating to obtaining Institutional Review Board (IRB) review and approval are met. I will promptly report to the IRB all changes in the research activity.

I have understood that the TBDx automated microscopy system is for investigational use only since the performance characteristics of this product are being established. As such, results from the TBDx will not be used for patient management or care.

I will maintain confidentiality of protocol and all other investigational materials.

I agree to ensure that all colleagues and employees assisting in the conduct of the study are informed about their obligations in meeting the above requirements.

____

Dr. Ngoc Lan Nguyen Thi Date

____

Dr. Eduardo Gotuzzo Date

4 BACKGROUND

Despite the recent progress in Tuberculosis (TB) diagnosis over the last few years, only countries with more developed laboratory capacity have access to culture and molecular methods. Sputum smear microscopy remains the most common method for diagnosing TB worldwide and is still used for case reporting classifications. However, smear microscopy is labour intense, requires extensive training to become proficient and interpretation of results can vary between readers, particularly in specimens with very few organisms.

Signature Mapping Medical Sciences, Inc. (Virginia, USA) has developed and trained software to detect *M. tuberculosis* (MTB) in digital images acquired from Auramine-O stained sputum smears using fluorescence microscopy (FM). TBDx is an innovative smear microscopy system that automatically loads slides onto a microscope, focuses and digitally captures images and then classifies smears as positive or negative using computerised algorithms.

A 2011 proof of concept study illustrated the potential of TBDx and at that time it achieved a sensitivity of 76%. However, specificity was far below routine microscopy and required improvement. Since 2011 important software changes were implemented and data from a more recent study looks promising. The performance of TBDx compared to culture showed a sensitivity of 74 to 80% and specificity ranging from 79 to 96% in a study conducted in South Africa.

Therefore FIND will assess the feasibility of the TBDx platform in high workload laboratories experienced in microscopy. The aim is to determine performance characteristics compared to culture as the reference standard and up to two other available automated microscopy platforms when available. Furthermore, we will determine the user appraisal and technical suitability in routine settings.

5 PURPOSE OF THE STUDY

The purpose of this study will be to determine the performance characteristics of TBDx with culture as a gold standard and to compare it with up to two other automated microscopy platforms when available. In addition, we will assess the laboratory technicians' appraisal and technical suitability of the TBDx system.

6 STUDY OVERVIEW

This will be a blinded, prospective study to determine the performance of the TBDx system for detection of pulmonary TB in comparison to LED microscopy and culture as a gold standard. The study will involve recruitment of 300 TB suspects per site with a high workload and experienced technicians. Participants will be recruited under the FIND TB Reference Materials (TBRM) project and leftover sputum samples used for the assessment of the TBDx.

The study is planned to start in late July 2014. Subjects' recruitment is expected to take approximately 3 months with final culture results available 2 months after the end of enrolment.

7 PRODUCT DESCRIPTION

The TBDx automated slide management and detection system (Signature Mapping Medical Sciences, Inc., a wholly owned subsidiary of Applied Visual Sciences, Inc., Virginia, USA; AVS) is able to detect MTB from digital

images acquired from Auramine-O stained sputum smears, using fluorescence microscopy. This automated platform is based on an Olympus BX41 microscope with a 40 x objective lens, fitted with an Olympus XC 10 colour camera and a movable slide stage, with an attached computer that receives high-quality digital images acquired from the camera. The computer then operates detection algorithms that segment, evaluate, and classify objects of interest in these images which can be stored for subsequent review. The platform is able to integrate an optional 200-slide automated slide loader for high volume settings. The application can capture 100, 300 or more digitals fields-of-view and can provide results for positive or negative smears in 5 minutes or less.

8 STUDY OBJECTIVES

Primary Objectives:

- To determine sensitivity, specificity and predictive values of TBDx among adults with suspicion of TB using culture as a gold standard.
- To compare the performance of TBDx to LED FM and to up to two other available automated microscopy platforms when available.
- To determine the feasibility of use of the TBDx system at a high workload reference laboratory.

Secondary Objectives:

- To determine minimal training needs.
- To assess the difference between direct vs. concentrated smear when using TBDx.
- To determine the performance of TBDx in combination with Xpert MTB/RIF (TBDx as a screening test).
- To assess the user appraisal regarding ease of use, hands-on-time and perceived benefit.
- To identify potential difficulties for implementation.

9 STUDY DESIGN

This will be a blinded, prospective study to determine the performance of the TBDx system in patients with symptoms of pulmonary TB (PTB) in comparison to conventional methodologies.

A. Training phase: left over sputum samples from TB suspects will be collected in advance. Duplicate smears will be prepared from direct and concentrated sputum. One set per subject will be used for characterization using LEDFM to avoid fading (photo bleach). The other set will be kept and stained with Auramine-O 24 hours before the training starts. The total number of samples for training will be 25 smear-positive and 25 smear-negative.

B. Feasibility study: sputum samples from TB suspects will be collected under the FIND TBRM project. For the current study two additional smears will be prepared from leftover material: one direct and one concentrated. Slides will be stained with Auramine-O and will be read with both LED FM and TBDx. The order of smear reading will be alternated and will follow a pre-established schedule. Both solid and liquid culture results from the same sample will be available and will be used as a gold standard.

9.1 Sample Size

A recent study conducted in South Africa determined that the sensitivity of TBDx versus culture was up 80% and the specificity was determined to be up to 96%. Furthermore, based on previous studies the local TB prevalence is roughly 45% in both Vietnam and Peru (referral centers for TB). Therefore, with 300 enrolees per site we expect to have 270 TB cases and at least 330 Non-TB cases. This will provide us with a sufficient number of patients in each group to establish the performance endpoints with relatively narrow confidence intervals [Li JL et al, Stat Med. 2004].

9.2 Study Timelines & Deliverables

The training and set-up visits for the participating sites are planned for July 2014 and will include one member of the FIND team and one AVS staff for study training and initial installation and trouble-shooting of the TBDx system.

Enrolment will start immediately after training and is expected to take up to three months. The last culture results will be ready two months after the end of enrolment.

Activity	Dates
IRB & import permit submission	Jun-14 to Jul-14
On-site training	Jul-14
Feasibility study recruitment	Aug-14 to Oct-14
Culture completion	Aug-14 to Dec-14
Report preparation/Publication	Jan-14

Table 1.*Study timelines.*

9.3 Study population

9.3.1 Inclusion criteria

- Persistent cough (≥2 weeks) and at least one other typical symptom of PTB (fever, night sweats, malaise, recent weight loss, contact with active case, hemoptysis, chest pain, loss of appetite)
- Provision of informed consent (FIND TBRM)
- Provision of sputum for adequate testing
- Patient aged 18 years or above

9.3.2 Exclusion criteria

- Patients receiving any anti-TB medication, in the 60 days prior to testing.
- Patients with only extra-pulmonary disease

9.4 Subject recruitment

Subjects who have symptoms consistent with PTB presenting to health facilities will be asked to participate in the FIND TB Reference Materials project. Participants may be identified by regular clinic staff or study personnel during an initial interview. They must be told that participation is voluntary and that they have the opportunity to ask questions individually. A consent form will be signed for the FIND TBRM project, leftover samples will be used for TBDx assessment and other automated microscopy platforms when available.

Eligible participants who signed the informed consent form will be asked to provide a sputum sample. HIV testing will be performed for all subjects under the FIND TB Reference Materials project. All results must be recorded in the Case Report Form (CRF), which is to be entered in the FIND database. Study participants with an incomplete CRF or inadequate samples must be withdrawn.

9.5 Sample processing

Sputum samples will be collected on the enrolment day when the patient is first seen. A member of the study team will provide the patient with a clean, single-use sputum cup and instruct the patient on how to provide sputum. The sputum cup should be labelled with the patient study ID before being given to the patient. Sputum will be transferred to the reference lab for testing. If necessary, sputum can be refrigerated overnight for next-day testing. Samples should be handled as infectious using established laboratory procedures and/or institutional guidelines. Sputum samples will be processed according to the following flow:

9.5.1 Sample Flow

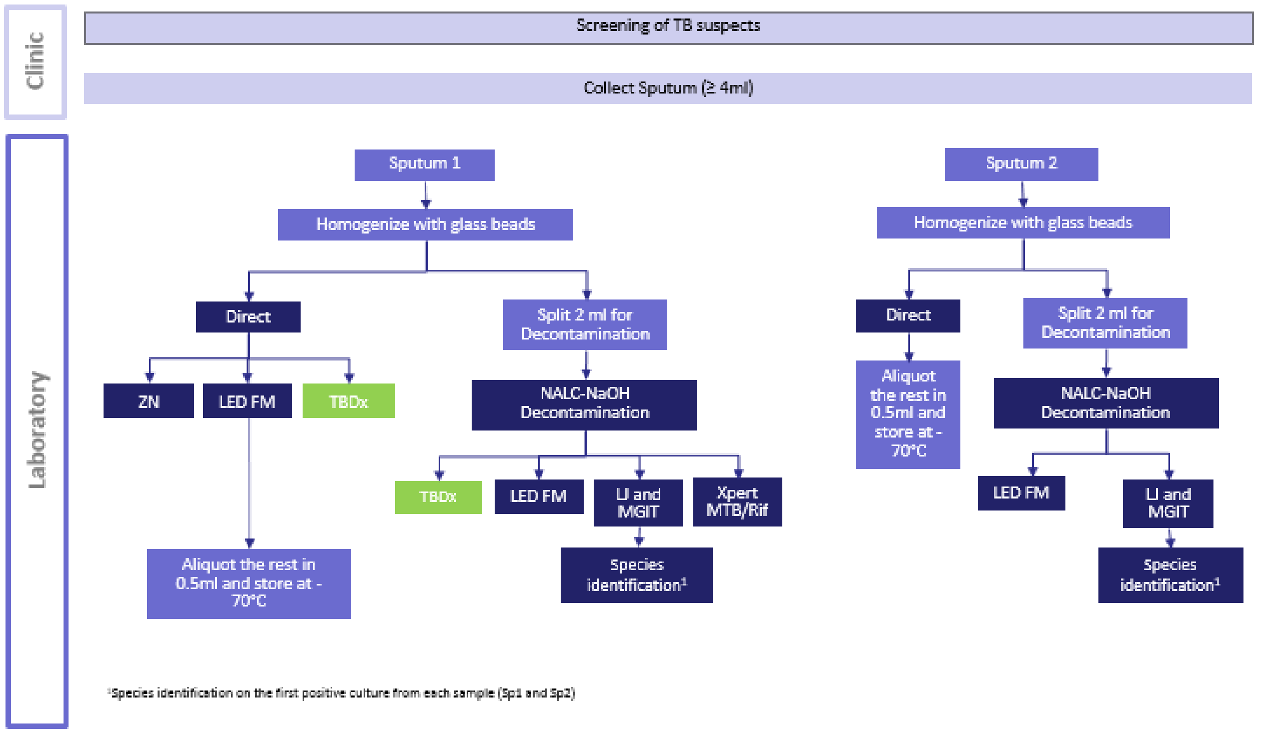

Figure 1*.Sample flow including FIND TBRM flow (sputum samples).*

10 TBDx test procedure

Slides stained with Auramine-O will be ready using the TBDx system. For the purposes of this study, slides will be loaded manually. Once the slide is placed on the microscope stage, TBDX automatically focuses the microscope, digitises 300 fields of view at 40X magnification and downloads these data to a computer, which then uses proprietary algorithms to detect and count AFBs on the digitised fields of view. Each of the 300 fields of view are then analysed by TBDx, regardless of the number of AFBs detected on previously examined fields of view for that smear. TBDx provides specific counts of AFB. Slides with no AFBs detected by TBDx are then classified as TBDx negative, 1-9 AFBs as TBDx scanty positive and ≥1 AFB as TBDx positive. The system allows the definition of categories for positivity and these will be set during training and maintained throughout the study.

The lab technician operating the TBDx will be blinded to all other results.

10.1 Laboratory Requirements

The TBDx will be setup close to where the LED microscope is located. No special requirements are needed in terms of space or ventilation.

10.2 Maintenance

If possible, the TBDx computer should be connected to the internet. This will enable remote troubleshooting. No maintenance is expected to be required for the TBDx system for the duration of the study; however, should any malfunctions arise, the sites should inform FIND immediately and FIND will provide advice or replace the instrument if necessary.

10.3 Waste Procedures

All patient residual specimens should be disposed of as per local institutional guidelines.

10.4 Methods to be compared, description of reference standard

10.4.1 Study method

- TBDx

10.4.2 Gold standard

- Löwenstein Jensen culture*
- MGIT culture*
- Confirmation of MTB in all pos cultures will be required (Capilia rapid test or conventional methods).

10.4.3 Comparative methods

- Smear microscopy of Auramine-O stained slides using LED FM*
- Xpert MTB/RIF (screening test scenario)
- Up to two other automated microscopy platforms when available

*As per FIND TBRM Laboratory Methods

10.5 Minimization of bias

Under the FIND TBRM project a consecutive series of patients with typical symptoms of TB will be included. Similar inclusion and exclusion criteria will therefore apply for this study.

In order to avoid bias due to photo bleach effect, slides will be read with both LED FM and TBDx and the order of smear reading will be alternated according to a pre-established schedule.

The TBDx results will be based on predefined algorithms. It will therefore not be necessary to blind the TBDx users against FM smear results. However, it will be necessary to blind the lab technicians involved in FM smear microscopy against all other test results (TBDx and Xpert MTB/RIF done as part of the FIND TBRM project).

11 SUPPLY AND INVENTORY CONTROL

All equipment and supplies for the entire duration of the study will be shipped to the sites before the beginning of the study. No additional shipments are planned.

It is the responsibility of the local Study Coordinator to maintain an updated inventory of the study materials and to inform FIND immediately if additional materials need to be shipped. An updated inventory should be shared with FIND at least once a month or at any other time upon request.

11.1 Shipments - Import and export permit needs

The sites must apply for the necessary import and export immediately on protocol submission.

Catalog Nr/ serial Nr	Description (as on the invoice for shipping & as will labelled on the package)	Qty	Shipped from	Country of Origin	Manufacturer - Country
TBD	Intel CPU Computer	1	Applied Visual Sciences, Inc. 525K East Market Street, #116 - Leesburg, VA 20176 USA –*to be handcarried by APVS*	USA	USA
TBD	Olympus XC10 Camera	1		Japan	Japan
TBD	Prior Z-Drive Motor	1		USA	USA
TBD	BH2 MV Power Transformer	1		Japan	Japan
TBD	Computer mouse, cables, halogen lamp	1		USA	USA
TBD	Olympus BX Microscope	1		Japan	Japan
TBD	Prior ES Stage	1		USA	USA
TBD	OSIS Joystick	1		USA	USA
TBD	MV Lamp Housing	1		Japan	Japan
TBD	Microscope cables, cover, observation mount	1		USA	USA
TBD	Glass slides	1000	TBD	South Africa	South Africa

Table 2*. Import and export permit needs.*

12 DATA MANAGEMENT AND QA

Paper case report forms (CRFs) as well as electronic CRFs via a web-based database for on-site double data entry will be provided by FIND for all conventional laboratory data. Remote training on data management, including how to enter data into the database will be conducted by FIND at the beginning of the study. Detailed instructions on how to fill out the CRFs will be provided during the initial training visit. Data must be entered in near-real time (e.g. less than a week of the data being collected or results obtained). Two data entry personnel should be employed at each site to perform first and second data entry separately. Second data entry will be used for the final data analysis. The sites are expected to outline a system for when data entry occurs for each part of the study process (e.g. enrolment, culture results, follow-up, etc.).

FIND will be able to review all data entered in real-time. Query and report functions will allow basic real-time data checking and data analysis. Changes in the database will be tracked automatically and all data will be backed up in real-time. FIND will conduct data checking and report back to sites for correction of missing or inconsistent data on a regular basis.

Any changes on a case report form once started will be documented and signed. Original CRFs will be stored on-site for at least 2 years. 10% of CRFs will be copied and sent to FIND to check the original data against that in the database to ensure quality of data and determine a final error rate.

When final culture results are available, the final data set will be locked for analysis final. The results of this trial are expected to be published under the supervision of FIND.

13 STUDY QA

13.1 Trial Site Certification and Proficiency Testing

According to FIND's ISO standards, the sites will need to undergo FIND certification of lab and clinic. This certification requires a trial site visit of a FIND laboratory expert prior to study initiation unless a certification has taken place less than 3 years before the study start.

13.2 Training, monitoring and auditing

FIND is the study sponsor and is responsible for planning, managing and monitoring the study. The site study team will receive technical on-site training by a member of FIND Clinical Team and a staff member of AVS during the initial set-up visit. Completion of case report forms (CRF) and data management will be part of the initial training. Staff at clinical enrolment sites will be trained in checking study inclusion criteria, obtaining informed consent for the FIND TBRM study and will be familiarized with the SOP for sputum collection by the on-site study team.

FIND will also appoint a Study Coordinator based at FIND office in Geneva. The sites will contact FIND Study Coordinator or a designated study monitor by e-mail or telephone if they have any difficulty or question. FIND Study Coordinator will intercede with AVS for any TBDx® related issues. In order to preserve the independence of the study, the sites should not contact AVS directly and all communication should be channelled through FIND. Telephone conferences between FIND and each site will be scheduled once a month or upon necessity. Monitoring site visits will be conducted by a member of FIND Clinical Team according to the site monitoring plan.

14 DATA ANALYSIS

The sensitivity, specificity and predictive values for TBDx in comparison to culture as the gold standard will be calculated. Additionally, we will compare the performance of TBDx to that of LED FM on both direct and concentrated smears. We will also assess the performance of TBDx in combination with Xpert MTB/RIF compared to culture (TBDx as a screening test scenario). A user appraisal and proficiency testing forms will be developed to assess the operational endpoints. Analysis will be done per specimen applying the following rules:

DIAGNOSIS	DESCRIPTION
LED FM smear-positive	≥ 1+ smear (≥20/40 fields) on LED FM<br>Smear positive with only negative or contaminated cultures will be excluded from analysis.
LED FM scanty-positive smear	1-19 AFB per 40 fields
Culture-positive	≥ 1 LJ and/or MGIT culture growth confirmed MTB complex.<br>Cross-Contamination: LJ culture with ≤ 20 colonies or MGIT culture with MTB growth ≥28 days will be excluded from analysis.<br>NTM: Specimens with growth of mycobacteria other than MTB complex only will be excluded from analysis.
Contaminated culture	LJ: Cultures completely overgrown by bacterial or fungal contaminations within 3 weeks (discarded). In case of mixed cultures, isolated MTB colonies transferred to new LJ tube (repeat culture).<br>MGIT: Instrument positivity w/o detection of AFB.<br>2 contaminated cultures (of 2) will lead to exclusion from analysis.
Non-TB case	Smear negative and culture-negative.
Indeterminate cases (excluded from main analysis)	Excluded from analysis as indeterminate cases will be patients with incomplete case report forms; smear-positive, culture-negative cases; cases with only contaminated cultures; and cases with Mycobacterium other than tuberculosis only.

Table 3: *Definitions for analysis.*

15 STUDY ETHICS

The sites will obtain local Institutional Review Board (IRB) approval and national IRB approval where appropriate.

Specimens will be collected, as required for routine diagnostic evaluation, from patients who are suspected of having pulmonary TB under the FIND TBRM project for which participants will signed an Informed Consent Form. Required clinical information will consist of medical history, clinical examination and chest radiography. HIV status will be assessed under the FIND TBRM project for which participants will signed a separate Informed Consent Form. Additional sputum smears will be prepared from leftover direct and concentrated sputum for TBDx. Results from the TBDx system will <u>not</u> be used for patient management or care.

15.1 IRB and approval procedure

A copy of this protocol will be submitted to IRB for approval before the beginning of the study. However, no specific informed consent will be sought for this study other than the FIND TBRM informed consent for provision of samples which covers testing of new diagnostic tests.

15.2 Possible benefits associated with the study

Patients participating in this study will already receive the best possible diagnostic work-up. Diagnosis will be accelerated by the use of automated liquid MGIT culture for case detection and rapid speciation with Capilia.

15.3 Risks associated with the study

There is no foreseen risk associated with this study.

15.4 Subject identification and confidentiality

Under the FIND TBRM project, all patient information will be treated in a strictly confidential manner and will be linked to a unique ID number and not to personal identifiers. CRF information will be entered in a password protected database and the same principles will apply to this study.

15.5 Confidentiality of study documents and subject records

Under the FIND TBRM project, study documents and patient CRFs will be regarded as strictly confidential and kept in a secure location with access restricted to study staff only. All subjects will be identified in the trial only by a code number and not by name or any other personal identifier and the same principles will apply to this study.

FIND APPROVAL

___________________________________	______________________
Study Coordinator	Date
___________________________________	______________________
Head of Program	Date
___________________________________	_______________________
CSO	Date
